# Supplementary material for: Tricine as a Novel Cryoprotectant with Osmotic Regulation, Ice Recrystallization Inhibition and Antioxidant Properties for Cryopreservation of Red Blood Cells
Source: Int J Mol Sci. 2022 Jul 30;23(15):8462. doi: 10.3390/ijms23158462 (PMC9369174; doi:10.3390/ijms23158462)
Supplement: Supplementary file 1 [file ijms-23-08462-s001.zip › Supplementary Materials-Figures Tables.pdf]

Supplementary Materials

Tricine as a Novel Cryoprotectant with Osmotic Regulation, Ice Recrystallization Inhibition and Antioxidant Properties for Cryopreservation of Red Blood Cells

Xiangjian Liu, Yuying Hu, Wenqian Zhang, Deyi Yang, Yuxin Pan, Marlene Davis Ekpo, Jingxian Xie and Songwen Tan *.

Xiangya School of Pharmaceutical Sciences, Central South University, Changsha, Hunan 410013, China.

Experimental Section

Analytic hierarchy process (AHP) model

In AHP model analysis, the safety, effectiveness and concentration were considered as criteria, while HES, glycerol and tricine were considered as plans. Saaty's fundamental scale [1], which contained nine levels of preference, was used to creat judgment matrixes of plans and criteria. The largest eigenvalue λ_{max} of judgment matrix was used to calculate the consistency index (CI) and the consistency ratio (CR) with the equation (S1) and (S2). The mean random CI was taken from Saaty's previous study [1]. If the CR was less than 0.1, the consistent of the matrix was considered to be acceptable. Then the eigenvector of λ_{max} was normalized as the weight vector. Therefore, the weight of plans to the objective could be obtained.

$$\mathrm{CI}=\frac{\lambda_{\max}-\mathrm{n}}{\mathrm{n}-1} \qquad \text{(S1)}$$

$$\mathrm{CR}=\frac{\mathrm{CI}}{\text{Mean random CI}} \qquad \text{(S2)}$$

Rank-Sum Ratio (RSR) model

In RSR model, trehalose, DMSO, HES and tricine were chosen as alternative CPAs, while thawed RBCs recovery, cost, optimal concentrations and biocompability were chosen as criteria. The RSR model was operated as following steps: First, an m×mn matrix **A** was created and denoted by matrix (S3) for initial analysis. m meant the number of alternative CPAs and n meant the number of criteria. Secone, the initial matrix **A** was converted to a rank matrix **R** by the equation (S4)-(S8). The thawed RBCs recovery and biocompability were considered as high-quality criteria, while cost and optimal concentrations were considered as low-quality criteria. Third, the RSR could be calculated by the equation (S7). Fourth, the *RSR*, frequency *f*, cumulative frequency $\sum f$,mean rank $\overline{R}$ and $\overline{R}/n\times100\%$ could be summarized, and the value of Probit could be obtained. Fifth, linear regression was performed between RSR and Probit, and R^2 was used as a factor to analyze fitting degree. Last, the CPAs could be classified

into different levels through Probit. The performance of each CPAs was proportional to its level [2].

$$\mathbf{A}=(a_{ij})_{\mathbf{m}\times\mathbf{n}}=\begin{pmatrix} a_{11} & a_{12} & \cdots & a_{1n} \\ a_{21} & a_{22} & \cdots & a_{2n} \\ \vdots & \vdots & \vdots & \vdots \\ a_{m1} & a_{m2} & \cdots & a_{mn} \end{pmatrix} \tag{S3}$$

$$\mathbf{R}=(R_{ij})_{\mathbf{m}\times\mathbf{n}}=\begin{pmatrix} R_{11} & R_{12} & \cdots & R_{1n} \\ R_{21} & R_{22} & \cdots & R_{2n} \\ \vdots & \vdots & \vdots & \vdots \\ R_{m1} & R_{m2} & \cdots & R_{mn} \end{pmatrix} \tag{S4}$$

where

$$R_{ij}=1+(n-1)\frac{x_{ij}-X_{\min}}{X_{\max}-X_{\min}}\text{, for high-quality criteria} \tag{S5}$$

and

$$R_{ij}=1+(n-1)\frac{X_{\max}-x_{ij}}{X_{\max}-X_{\min}}\text{, for low-quality criteria} \tag{S6}$$

and

$$X_{\max}=\max(x_{1j},\ x_{2j},\ \ldots x_{mj}) \tag{S7}$$

and

$$X_{\min}=\min(x_{1j},\ x_{2j},\ \ldots x_{mj}) \tag{S8}$$

$$RSR_{\mathrm{i}}=\frac{\sum_{j=1}^{\mathrm{n}} R_{ij}}{m\times n} \tag{S9}$$

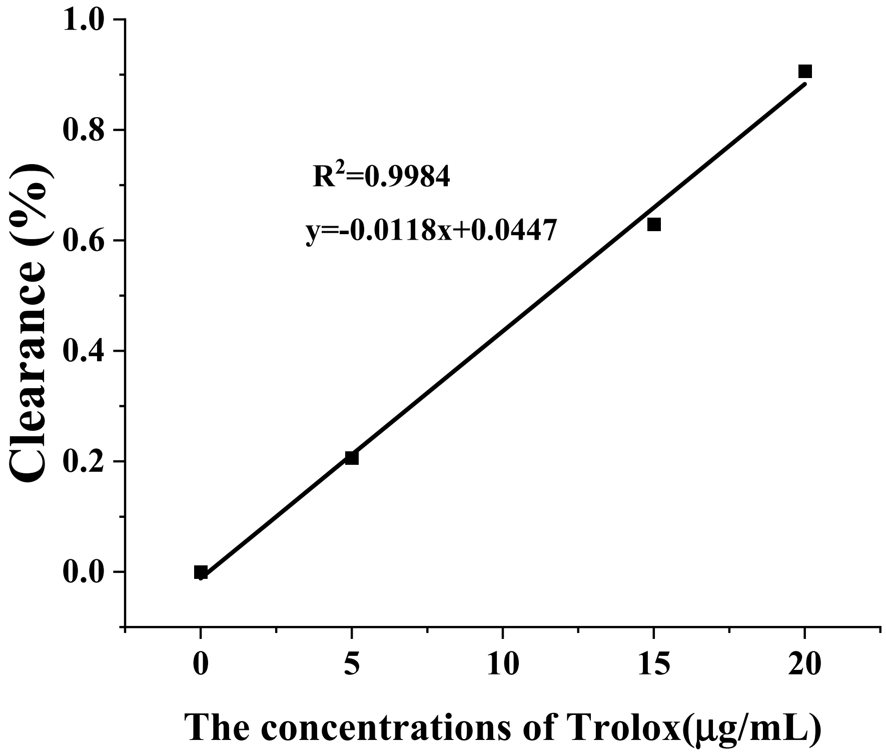

Figure S1. The standard curve of clearance and concentrations of Trolox.

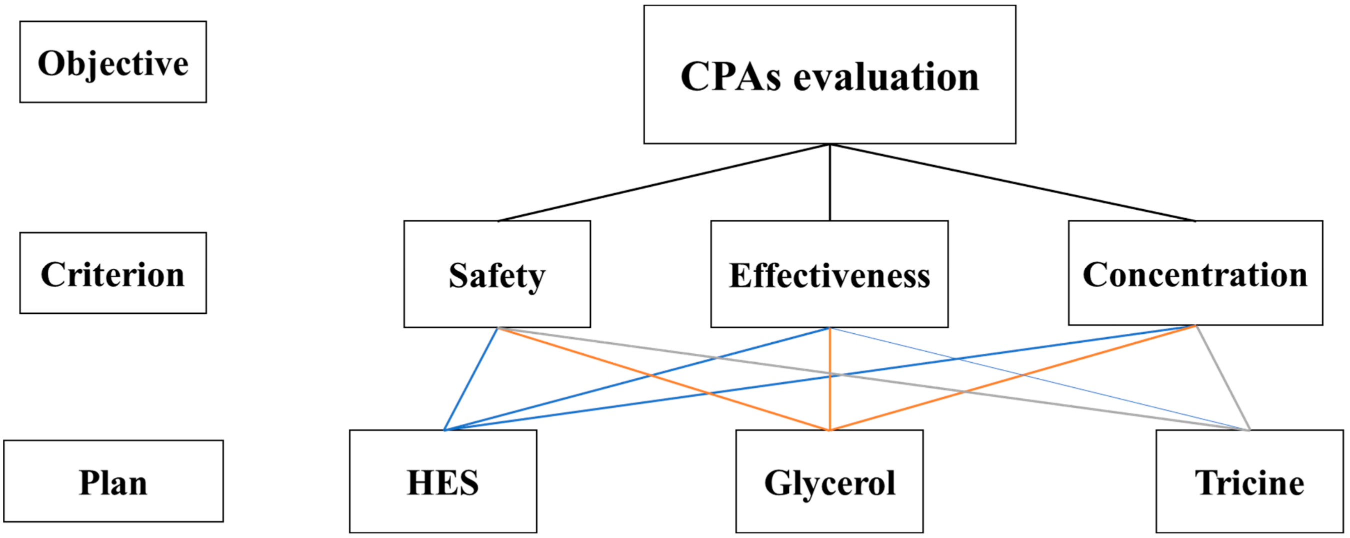

Figure S2. Analytic hierarchy process model of CPAs evaluation.

Table S1. Saaty's fundamental scale

Numerical scale	Verbal scale
1	Equal importance
3	Moderate importance
5	Essential importance
7	Very strong importance
9	Extreme importance
2, 4, 6, 8	Intermediate values

Table S2. Results of pairwise comparisons among criteria (CR=0.009).

Criteria	Safety	Effectiveness	Cost	Weight
Safety	1	1	4	0.457
Effectiveness	1	1	3	0.416
Concentration	1/4	1/3	1	0.126

Table S3. Results of pairwise comparisons among plans (Safety).

Safety (CR=0.009)				
	Glycerol	HES	Tricine	Weight
Glycerol	1	1/3	1/2	0.164
HES	3	1	2	0.539
Tricine	2	1/2	1	0.297

Table S4. Results of pairwise comparisons among plans (Effectiveness).

Effectiveness (CR=0.018)				
	Glycerol	HES	Tricine	Weight
Glycerol	1	4	2	0.557
HES	1/4	1	1/3	0.123
Tricine	1/2	3	1	0.320

Table S5. Results of pairwise comparisons among plans (Concentration).

Effectiveness (CR=0.009)				
	Glycerol	HES	Tricine	Weight
Glycerol	1	1/2	1/3	0.164
HES	2	1	1/2	0.297
Tricine	3	2	1	0.539

Table S6. The weight of plans to the objective.

Plans	Weight	Rank
Glycerol	0.316	3
HES	0.334	2
Tricine	0.350	1

Table S7. The original data and rank in RSR model.

Types of CPAs	Concentrations (%wt)^{b}		Thawed RBCs recovery (%)		Biocompability (%)	
	x_1	R_1	x_2	R_2	x_3	R_3
Glycerol	20.0	1.0	89.5	3.0	74.9	1.0
HES	13.0	2.0	39.7	1.0	97.5	3.0
Tricine	6.0	3.0	81.2	2.7	95.3	2.8

Table S8. The indexes of RSR model.

CPAS	RSR	f	Σ f	$\overline{R}$	$\overline{R}/n\times100\%$	Probit	Linear regression equation	R^2
Glycerol	0.5556	1	1	1.0	33.3	4.569		
HES	0.6667	1	2	2.0	66.7	5.431	RSR=-0.440+0.212*Probit	0.957
Tricine	0.938	1	3	3.0	91.7	6.383		

[a]This data was corrected by the equation: $(1-\frac{1}{4n})$

Table S9. The divided levels of CPAs.

Levels	Threshold of Probit	CPAs
Medium	< 4	/
Good	4~6	Glycerol and HES
Excellent	6~	Tricine

[1] T.L. Saaty, J.P. Bennett, A theory of analytical hierarchies applied to political candidacy, Behavioral Science 22(4) (1977) 237-245.http://doi.org/10.1002/bs.3830220402

[2] Z. Wang, S. Dang, Y. Xing, Q. Li, H. Yan, Applying Rank Sum Ratio (RSR) to the Evaluation of Feeding Practices Behaviors, and Its Associations with Infant Health Risk in Rural Lhasa, Tibet, Int. J. Environ. Res. Public Health 12(12) (2015) 15173-81.http://doi.org/10.3390/ijerph121214976
